# Role of Dynamic Contrast-Enhanced MRI in Detecting Post-Treatment Local Recurrence of Soft-Tissue Sarcomas: A Systematic Review and Meta-Analysis

**DOI:** 10.3390/diagnostics16010136

**Published:** 2026-01-01

**Authors:** Arash Azhideh, Howard Chansky, Peyman Mirghaderi, Sara Haseli, Bahar Mansoori, Navid Faraji, Chankue Park, Shakiba Houshi, Majid Chalian

**Affiliations:** 1 Department of Radiology, Division of Musculoskeletal Imaging and Intervention, University of Washington, Seattle, WA 98105, USA; peymanm@uw.edu (P.M.); shasel@uw.edu (S.H.); chankue@uw.edu (C.P.); s.houshi@yahoo.com (S.H.); mchalian@uw.edu (M.C.); 2 Department of Orthopaedic Surgery, University of Washington, Seattle, WA 98105, USA; chansky@uw.edu; 3 Department of Radiology, Division of Abdominal Imaging, University of Washington, Seattle, WA 98105, USA; mansoori@uw.edu; 4 University Hospitals Cleveland Medical Center, Case Western Reserve University School of Medicine, Cleveland, OH 44106, USA; navid.faraji@uhhospitals.org

**Keywords:** MRI, sarcoma, soft-tissue sarcoma, dynamic contrast

## Abstract

**Background**: The role of dynamic contrast-enhanced magnetic resonance imaging (DCE-MRI) in detecting soft-tissue sarcoma (STS) local recurrence (LR) following therapeutic intervention was evaluated. **Method**: PubMed, Embase, and Scopus were systematically searched from January 1990 to 1 February 2024 for studies evaluating DCE-MRI for LR detection in histologically confirmed STS following surgery. Two independent reviewers screened studies and extracted data, and a bivariate diagnostic test accuracy meta-analysis was performed to estimate pooled sensitivity, specificity, and the area under the summary receiver operating characteristic (SROC) curve. **Results**: Six studies, including 309 patients (110 with LR and 199 without LR), met the inclusion criteria. Across studies, DCE-MRI qualitative features (such as early rapid arterial enhancement and malignant time–intensity curves) and quantitative or semiquantitative parameters (such as volume transfer constants [Ktrans and Kep], initial area under the curve [iAUC], and relative plasma flow [rPF]) consistently differentiated LR from post-treatment change. When DCE-MRI parameters were added to conventional MRI, the pooled sensitivity and specificity for LR detection were 98% and 83%, respectively, with an SROC area under the curve of 0.94, indicating high overall diagnostic accuracy. **Conclusions**: DCE-MRI increases the accuracy of LR detection when combined with conventional MRI and offers a higher specificity and sensitivity in distinguishing LR from post-surgical changes, which support consideration of adding DCE-MRI when LR is suspected; prospective standardized studies are warranted.

## 1. Introduction

Soft-tissue sarcomas (STSs) are a rare but clinically significant group of mesenchymal neoplasms, accounting for less than 1% of adult malignancies [1]. Despite multimodal therapeutic strategies in the management of STS, the risk of local recurrence (LR) remains significant [2]. Risk factors for LR include patient age, tumor size, histological subtypes, absence of pre-operative radiotherapy, and positive surgical margins [3]. The incidence of LR has been documented to range from 6.5% to approximately 50%, with an average of about 20% [4]. Early detection of LR is critical to reduce morbidity and the need for extensive re-excisions and the development of further metastasis. Distinguishing LR from post-operative changes, such as scar tissue or fibrosis, poses a diagnostic challenge due to overlapping features in conventional MRI [5,6]. The complete absence of high signal intensity in the surgical bed in fluid-sensitive images has been suggested to be an excellent sign to exclude tumor recurrence, although LR might occasionally present with a hypointense signal in fluid-sensitive sequences. Other features like architectural distortion and signal intensity in both T1- and T2-weighted imaging are not typically distinguishing [5,6]. Functional MR techniques like dynamic contrast-enhanced MRI (DCE-MRI) have shown potential in improving diagnostic accuracy by providing additional qualitative and quantitative data regarding the pattern of enhancement, tissue vascularity, and perfusion based on time–intensity curves (TICs) [7,8].

In this systematic review and meta-analysis, we critically review the existing literature to assess the efficacy of DCE-MRI in detecting STS LR following surgical treatment.

## 2. Materials and Methods

### 2.1. Search Strategy

This systematic review and meta-analysis adhered to the Preferred Reporting Items for Systematic Reviews and Meta-Analyses (PRISMA) guidelines [9] (Table S1). Its objective was to evaluate the efficacy of DCE-MRI in identifying recurrent STS in post-treatment patients. This review extensively searched major databases, including PubMed, Scopus, Web of Science, Embase, and Google Scholar, starting from their inception, and without any limitations. We searched these databases from 1990 to 1 February 2024. The search strategy was crafted employing Medical Subject Headings (MeSHs) alongside additional search terms using the Boolean operators “AND” and “OR” with a combination of keywords, such as “soft tissue sarcoma”, “MRI”, “dynamic contrast”, “recurrence”, and their alternatives.

### 2.2. Study Selection

Two postdoctoral researchers with 3 and 5 years of experience independently screened the titles and abstracts identified in the search, followed by retrieval and evaluation of the full texts. The Patient, Intervention, Comparison Group, Outcome, and Study (PICOS) design was applied to select studies that met the following criteria: a. all patients diagnosed with STS by pathology and imaging findings; b. all patients who underwent surgery as treatment for STS; and c. all studies that used DCE-MRI in the follow-up period to assess the accuracy of detecting LR.

Studies were excluded if they used imaging modalities other than DCE-MRI, included fewer than ten patients in the study cohort (to avoid imprecise and unstable diagnostic estimates from very small series), or did not adequately report study endpoints. Animal studies, duplicate studies, conference abstracts, editorials, brief reports, book chapters, and dissertations were excluded.

### 2.3. Data Extraction

The same reviewers extracted data using standardized data abstraction templates designed with commercially available software (Excel, Microsoft, Redmond, WA). Disagreements at each screening or data extraction stage were first discussed to reach consensus between the two reviewers; if consensus could not be reached, a third reviewer (a fellowship-trained, board-certified radiologist with 9 years of experience) acted as an arbitrator. The data elements extracted from the included studies were as follows: the first author’s name, publication year, region, patient characteristics like age, gender (male/female), and DCE-based parameters, sample size (case/control), follow-up period, and performance metrics consisting of sensitivity and specificity.

### 2.4. Statistics

A bivariate diagnostic test accuracy (DTA) meta-analysis was conducted to evaluate the pooled sensitivity, specificity, and the summary receiver operating characteristic (SROC) curve. STATA version 14, using the MIDAS command, was employed to calculate the DTA parameters. This command uses a bivariate model that simultaneously considers both sensitivity and specificity.

All studies that reported sensitivity, specificity, case sample size, and control sample size, or provided data from which these metrics could be calculated, were included in this meta-analysis. These data were used to calculate true positives, false positives, true negatives, and false negatives, with the resulting values rounded to the nearest integer. The Cochran-Q test and I^2^ index were applied as indicators of heterogeneity within the meta-analysis, with significant heterogeneity defined as an I^2^ greater than 50% or a Cochran-Q test *p*-value < 0.10.

### 2.5. Quality Assessment

For the quality assessment of the included studies, we used the QUADAS-2 (Quality Assessment of Diagnostic Accuracy Studies 2) tool, a widely recognized instrument for evaluating the diagnostic accuracy of studies. It provides a structured approach for assessing the risk of bias and applicability concerns across four key domains: patient selection, index test, reference standard, and flow and timing (Table 1)

## 3. Results

### 3.1. Literature Search

**Figure 1:**
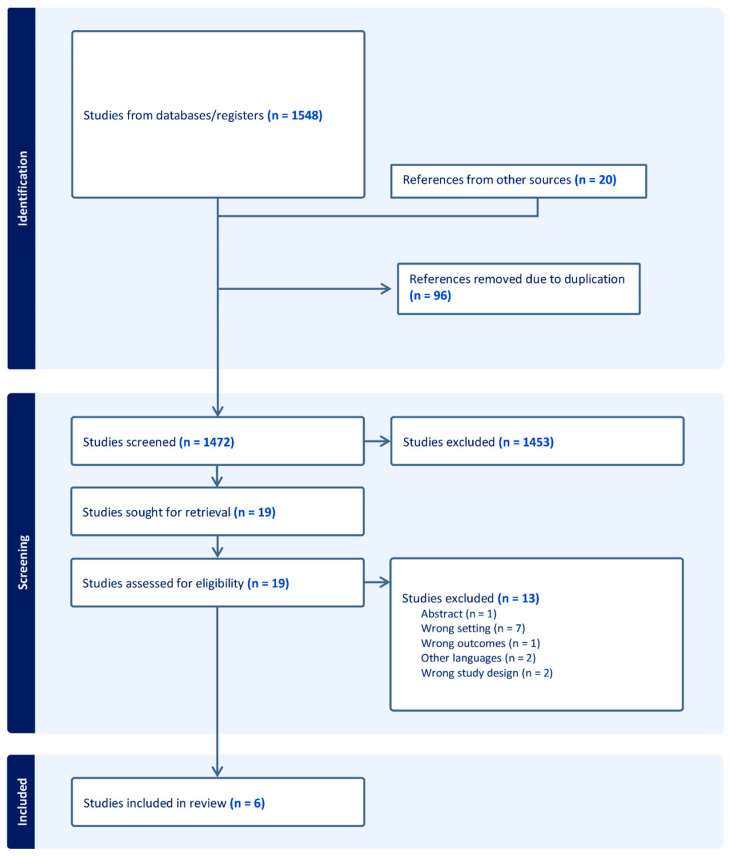
Flow diagram of the study selection process. Preferred Reporting Items for Systematic Reviews and Meta-Analyses (PRISMA).

The initial literature search identified 1548 studies, of which 96 studies were removed due to duplication. The remaining 1452 studies underwent screening of titles and abstracts, resulting in the exclusion of 1446 studies for reasons such as non-peer-reviewed status (e.g., conference abstracts and poster presentations), review articles, incomplete outcomes, studies involving patients with different pathologies, publications in languages other than English, or different outcome measurements. Ultimately, six unique peer-reviewed scientific studies met the inclusion criteria. The selection process for these studies is illustrated in Figure 1, which includes a flow diagram detailing the study selection methodology.

### 3.2. Patients’ Characteristics, STS Types, and Study’s Endpoint

The six studies were [5,7,10,11,12,13]. The study cohort, comprising 309 unique STS patients—ranging between 4 and 84 years old—[150 male (53%), 133 female (47%), and 26 (4.3%) unreported], included 110 patients in the case group and 199 in the control group. More characteristics of patients are provided in Table 2. Although different imaging sequences and features were investigated in each study, and not all included studies reported the accuracy of conventional MRI, all of them reported the sensitivity and specificity of DCE-MRI in detecting STS LR. Furthermore, several sequences with different protocols were used to assess the LR, with detailed information provided in Table 3.

Additionally, this review assessed the frequency of recurrent tumors in DCE-MRI in patients diagnosed with various STS types. The most frequently observed STS type across these studies was pleomorphic sarcoma (*n* = 44), followed by liposarcoma (*n* = 36), myxofibrosarcoma (*n* = 24), undifferentiated pleomorphic sarcoma (malignant fibrous histiocytoma) (*n* = 20), rhabdomyosarcoma (*n* = 10), malignant peripheral nerve sheath tumor (MPNST) (*n* = 8), and spindle cell sarcoma (*n* = 6). Less commonly observed types included myxoid liposarcoma (*n* = 4), chondrosarcomas (*n* = 3), and fibrosarcoma (*n* = 3).

### 3.3. DTA Meta-Analysis

**Figure 2:**
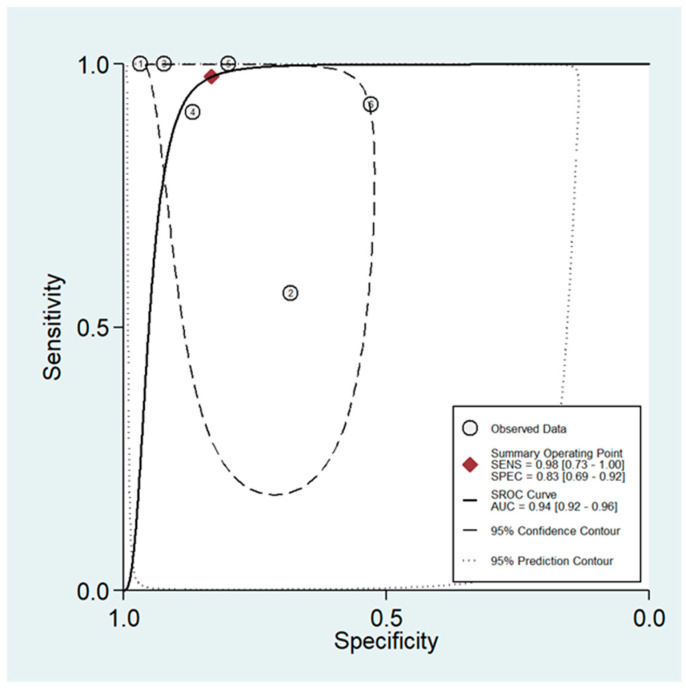
Summary receiver operating characteristic (sROC) curve depicting the diagnostic performance of dynamic contrast-enhanced magnetic resonance imaging (DCE-MRI) in detecting LR of STS post-treatment. The area under the curve (AUC) of the sROC is 0.94, indicating high diagnostic accuracy. The 95% confidence region is shown around the summary point, while a larger 95% prediction region highlights the substantial heterogeneity across studies, reflecting variability in diagnostic performance.

**Figure 3:**
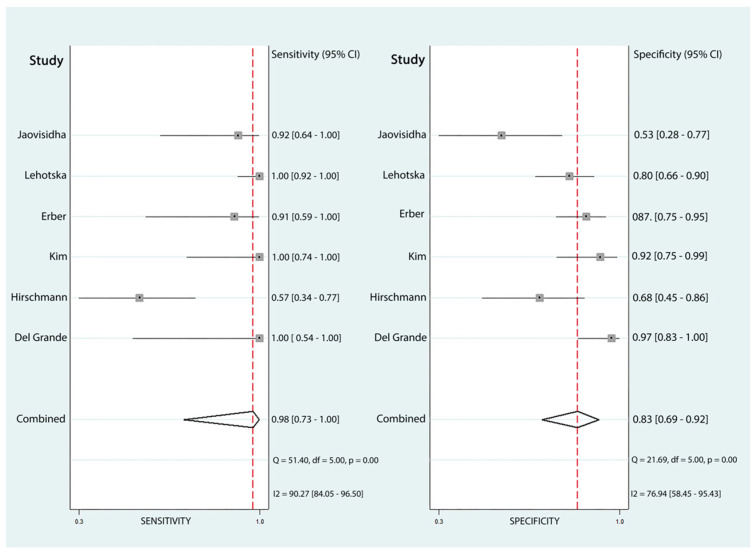
Bivariate model forest plot illustrating the pooled sensitivity and specificity of dynamic contrast-enhanced magnetic resonance imaging (DCE-MRI) for diagnosing LR of STS post-treatment.

Six papers with a total of 309 participants were included in this meta-analysis. The pooled sensitivity was 98% (95% CI: 73% to 100%), and the specificity was 83% (95% CI: 69% to 92%). Additionally, the area under the curve (AUC) for the SROC was 0.94 (95% CI: 0.92 to 0.96) out of 1, indicating the high accuracy of DCE-MRI in detecting LR of STS following surgical intervention (Figure 2). The forest plot of included studies evaluating the sensitivity and specificity of DCE-MRI is depicted in Figure 3. However, the heterogeneity of this analysis was high, potentially due to differences in the populations studied, variations in MRI readers, and the use of different imaging protocols and devices.

## 4. Discussion

This systematic review and meta-analysis evaluated the role of DCE-MRI in detecting LR of STS after surgical intervention, with an emphasis on its diagnostic accuracy and clinical applications. We found that, despite variations in the methods of DCE-MRI used, it demonstrates high sensitivity (98%) and specificity (83%) for LR detection. This suggests that DCE-MRI could potentially be one of the optimal methods for screening LR in cases of STSs. Although conventional MRI is often adequate for the initial evaluation of STS, it has limitations in LR detection, particularly in the early stages following neoadjuvant therapy [5,14,15]. Differentiating post-surgical changes, such as granulation tissue formation and fibrosis/scarring, from residual or recurrent tumors can be challenging [16,17,18,19]. In T1-weighted images, LR characteristically presents as hypointense areas of architectural distortion resembling scarring. Abnormal signal intensity in T2-weighted images may persist in the surgical bed for extended periods due to postsurgical fibrosis/scarring and/or LR. This distinction can be even more challenging when post-operative changes appear mass-like, with nodular areas greater than 5 mm or intramuscular architectural distortion, mimicking LR [5,11,20].

In the broader context of functional oncologic imaging, DCE-MRI provides perfusion and permeability information that is complementary to other advanced techniques such as diffusion-weighted imaging (DWI) and hybrid PET/MRI. DWI interrogates tissue cellularity via apparent diffusion coefficient (ADC) maps and may help distinguish a hypercellular recurrent tumor from predominantly fibrotic post-operative change, while PET/CT or PET/MRI can depict metabolically active disease and simultaneously assess for distant metastases [16]. However, DWI can be limited by susceptibility artifacts and heterogeneous post-operative tissue, and PET-based techniques are less widely available and associated with higher cost and exposure to ionizing radiation. DCE-MRI can be integrated into routine follow-up MRI protocols without additional radiation and, when combined with DWI as in the study by Erber et al., may further increase confidence in distinguishing LR from benign post-operative findings [8,16].

Del Grande et al. retrospectively studied 37 patients with histologically confirmed recurrent STS, most of which were high-grade sarcomas. Morphological characteristics with conventional MRI and the presence or absence of arterial enhancement in DCE-MRI using a time-resolved 3D volumetric sequence were recorded. The study reported a sensitivity of 100% and a specificity of 97% when DCE-MRI was added to conventional MRI. Their study demonstrated a high false-positive rate of conventional MRI in the false detection of LR, leading to nearly half of the cases without recurrence undergoing unnecessary biopsy of the post-operative scar. The authors concluded that DCE-MRI could significantly reduce the false-positive rate and unnecessary tissue sampling [5,15,21].

Both qualitative and quantitative methods can be used to assess tumor vascularity and the adjacent soft tissues. The presence of early arterial enhancement within the first seconds, which is defined by contrast enhancement of the nearby artery, is a common qualitative metric for assessment of STS LR [12,22,23,24,25]. Quantitative methods measure the pharmacokinetics of specific areas using parameters like the shape of the signal intensity curve, time to peak, and pharmacokinetic (PK) contrast enhancement [7,10,11,13,26,27].

Erber et al. prospectively and quantitatively evaluated the diagnostic accuracy of DCE-MRI in detection of LR in post-treatment extremity STSs. They used parameters such as relative plasma flow (rPF) and relative mean transit time (rMTT); their study revealed that rPF was significantly higher in LR compared with expected post-op changes, which makes it a strong indicator of LR [10,28].

Hirschmann et al. studied the role of MRI in detection of clinically silent LR; they focused on the findings of DCE-MRI and knowledge of surgical margins in identifying recurrence prior to the gross appearance of confirmed LR. They conducted a retrospective analysis comparing pre-operative MRI characteristics (both conventional and DCE) of 23 patients with post-treatment MRI taken before confirmed LR. The analysis consisted of morphological assessment in conventional MRI, and enhancement onset, dynamic enhancement pattern, and progression of enhancement type in DCE-MRI. A second analysis assessed LR visibility in post-operative MRI and subsequent third MRI to identify causes of false positives and false negatives. Since the majority of LR lesions were nodular, hyperintense, homogeneous in fluid-sensitive images, and centrally located in the surgical bed, conventional MRI was able to correctly detect 52% of patients, while one additional diagnosis was enabled by DCE-MRI. They also demonstrated that early enhancement—within six seconds—emerged as the most accurate imaging characteristic, which can improve the diagnostic accuracy of DCE-MRI. However, enhancement observed 5–7 months post-operatively may reflect persistent tissue remodeling secondary to increased vascularity [11,29].

Lehotska et al. evaluated the reliability of DCE-MRI in the detection of post-treatment LR of STSs in 95 patients. Post-contrast images were assessed in patients with diffuse hyperintensity in T2-weighted images (T2WIs) without a discrete mass. DCE-MRI was performed, followed by the subtraction method, and time–intensity curves (TICs) were analyzed. Biopsies were performed on 55 patients, including those with TIC types III, IV, and V (47 patients), and 8 patients with TIC type II. Among the 47 patients with TIC types III, IV, and V, 45 confirmed LR, while 2 demonstrated hypervascular granulation tissue. All eight patients with TIC type curve II were negative for recurrence in histology. DCE-MRI demonstrated a sensitivity of 100%, specificity of 80%, positive predictive value of 95.7%, and negative predictive value of 100%. These results indicate that DCE-MRI can accurately detect LR in patients based on TIC types [7]. DCE-MRI can provide detailed information about tissue characteristics by measuring parameters such as the volume transfer constant (Ktrans), rate constant (Kep), and initial area under the concentration curve (iAUC). Adding these parameters to conventional MRI has been shown to make DCE-MRI more reliable than conventional MRI alone in detecting STS LR [13,30].

This study is limited by sample size due to the rarity of STS and LR, which restricts the power of our analyses. In addition, our a priori decision to exclude very small case series with fewer than 10 patients may have further reduced the number of eligible studies, but was intended to minimize imprecision and potential small-study effects. Further studies comparing different parameters in the same cohorts with consistent results are required for validation and to increase the use of DCE-MRI in evaluating the LR of STS.

## 5. Conclusions

Conventional MRI has limitations in differentiating LR from post-surgical changes due to overlapping imaging features and technical limitations of the routine sequences. The accuracy of LR detection can be improved by combining conventional MRI with DCE-MRI. In our meta-analysis, adding DCE-MRI parameters to conventional MRI increased the pooled sensitivity and specificity to 98% and 83%, respectively. Among the parameters reported in the included studies, early arterial enhancement, TIC types III–V, higher Ktrans/Kep/iAUC values, and elevated rPF appeared to have the greatest clinical potential for distinguishing LR from expected post-treatment change. Future prospective studies should aim to standardize DCE-MRI acquisition (e.g., contrast administration protocol and temporal resolution) and analysis (e.g., pharmacokinetic modeling, ROI placement, and parameter thresholds) so that these metrics can be harmonized across institutions and translated into reproducible diagnostic criteria for routine clinical practice.

## Figures and Tables

**Table 1:** Quality assessment of included studies.

First Author	Year	Patient Reference	Index Test	Reference Standard	Flow and Timing
Kim et al. [10]	2016	Low risk	Low risk	Low risk	Low risk
Del Grande et al. [5]	2014	Low risk	Low risk	Low risk	Low risk
Jaovisidha et al. [11]	2011	High risk	Low risk	Low risk	Low risk
Hirschmann et al. [9]	2020	Low risk	Low risk	Low risk	Low risk
Erber et al. [8]	2022	Low risk	Low risk	Low risk	Low risk
Lehotska et al. [6]	2013	Low risk	Low risk	Low risk	Low risk

**Table 2:** Demographic information of the included studies.

First Author	Country	Confirmation Type	Study Group (LR Group)	Control Group (No LR Group)	Gender (M/F)	Age (Mean)	F/U Period (Months)
Del Grande [5] (2014)	USA	Bx/resection	6	31	LR: 3/3	71.3	15
					No LR: 14/17	55.7	
Erber [10] (2022)	Germany	Surgery	11	53	LR: 7/4	67	27.1
					No LR: 30/23	62	28.9
Hirschmann [11] (2020)	The Netherlands	Bx/resection	23	22	LR: 12/11	59.7	30
					No LR: 15/7	55	26
Kim [12] (2016)	Korea	Re-excision	12	26	LR: 7/5	49.7	1.5
					No LR: 9/17	50.8	1.5
Del Grande [5] (2014)	USA	Bx/resection	6	31	LR: 3/3	67	15
					No LR: 14/17	62	
Lehotska [7] (2013)	Slovakia	Biopsy	45	50	LR: 27/18	NR	NR
					No LR: 27/23	Median age for males: 51 Median age for females: 53	
Jaovisidha [13] (2011)	Thailand	Bx/resection	13	17	14/16	45.81	At least 2

NR: not reported; LR: local recurrence; F/U: follow-up; M: male; F: female.

**Table 3:** Specificity and sensitivity of the MR sequence in detection of STSs’ local recurrences.

Author	DCE Parameters	Sensitivity	Specificity	Classifications and Patterns of Enhancement
Erber [10] (2022)	Quantitative DCE-MRI parameters: rPF and rMTT	90.9% Optimum cutoff (rPF value of 6.74)	86.8% Optimum cutoff (rPF value of 6.74)	rPF was significantly higher in cases of local recurrence, while rMTT was slightly lower in local recurrence. Compared with morphological assessment, rPF had distinctly higher specificity and true positive value in detection of LR.
		72.7% Optimum cutoff (rMTT value of 0.61)	77.4% Optimum cutoff (rMTT value of 0.61)	
Hirschmann [11] (2020)	Start of enhancement in seconds relative to the start of enhancement in a nearby artery, pattern of dynamic enhancement (rim-like or internal/focal), and progression of enhancement type	57%	72%	Progression of enhancement type I + II (no enhancement or gradual increase in enhancement) was defined as benign patterns and that of type III–V (rapid initial enhancement, followed by a plateau/washout phase/sustained late enhancement) as malignant. In both patients with LR and those in the control group, most masses were nodular, hyperintense, and homogeneous in fluid-sensitive sequences and were centrally located within the surgical bed. Other imaging features were less common and did not differ significantly between the groups. Among all imaging criteria, early enhancement (<6 s) was the most accurate parameter.
Kim [12] (2016)	Quantitative parameters: Volume transfer constant between blood plasma and extracellular/EES (K_trans_) Rate constant between EES and blood plasma (K_ep_) * Volume of EES space per unit volume of tissue (V_e_) iAUC	100% (applied TCC-type threshold)	96%	Seven TCC patterns defined in DCE imaging: type I (continuously rapid increase in concentration over time); type II (gradual increase in concentration over time); type III (an initial rapid rise followed by a slower increase); type IV (an initial rapid rise followed by a plateau); type V (an initial rapid rise followed by a mild decrease); type VI (an initial rapid rise followed by slow washout); and type VII (an initial rapid rise followed by rapid washout). K_trans_, K_ep_, and iAUC were found to be significantly associated with the presence of residual tumor (*p* < 0.05).
		100% (DCE parameter threshold)	92%	
Del Grande [5] (2014)	Arterial enhancement pattern in maximum intensity projection and time–signal intensity curves	100%	97%	Recurrent tumors are characterized by early and rapid arterial phase enhancement in DCE MR imaging, whereas post-treatment inflammation and fibrosis enhance gradually over time. Time-resolved DCE MR sequence (only 5 extra minutes of imaging) is recommended to increase the specificity for the detection of recurrent disease.
Lehotska [7] (2013)	Perfusion TIC * ROI technique based on dynamic analysis and graphical expression of the time-dependent enhancement values with obtaining TIC to obtain tissue vascularization, perfusion, capillary permeability, and size and composition of interstitial space.	100%	80%	Patients were classified according to five types of TIC: No enhancement (TIC I, scar, fibrosis); Gradual enhancement (TIC II, inflammation, hyperemia, organizing hematoma); Rapid enhancement with plateau (TIC III, suspicious recurrence, biopsy required); Rapid enhancement with washout (TIC IV, high suspicion, biopsy); Rapid enhancement with sustained later increase (TIC V, high suspicion, biopsy); TIC types III, IV, and V are highly suspicious for local tumor recurrence and warrant percutaneous image-guided biopsy. Type II typically represents a pseudomass, and biopsy should be reserved for selected cases with an increased risk of recurrence, based on a multidisciplinary assessment. Active tumors typically demonstrate rapid early-phase enhancement during vascularization, whereas post-treatment changes tend to enhance later, usually after 2 min or more. Lesions with TICs demonstrating a gradual rise in enhancement (suggesting low perfusion and vascularization) can be managed with routine surveillance, whereas those showing an early, rapid increase in enhancement (indicating high perfusion and vascularization) should undergo biopsy and appropriate treatment.
Jaovisidha [13] (2011)	SS ratio between the artery and the lesion	92.31% (cut-off point ≥ 5.07)	54.55% (cut-off point ≥ 5.07)	The lesions with SS ratio > 9.28 were all benign at follow up of at least two months, whereas those with SS ratio < 1.05 were all recurrent tumors proven by biopsy or surgery. Overlapping SS ratios > 1.05 but < 9.28: the chance of having LR was approximately two and five times higher in patients with ratios of 5.07 and 1.55. The lower the value of the ratio, the greater the possibility of being a recurrent tumor.
		46.15% (cut-off point ≥ 1.54)	90.91% (cut-off point ≥ 1.54)	

MR: magnetic resonance; STS: soft-tissue sarcoma; DCE: dynamic contrast enhancement; LR: local recurrence; EES: extravascular space; TTC: time–concentration curve; TIC: time-to-intensity curve; rPF: relative plasma flow; rMTT: relative mean transit time; iAUC: initial area under the concentration curve; ROI: region of interest; SS: steepest slope. * (K_ep_ = K_trans_/V_e_).

## Data Availability

No new data were created or analyzed in this study. Data sharing is not applicable to this article.

## Supplementary Materials

The following supporting information can be downloaded at https://www.mdpi.com/article/10.3390/diagnostics16010136/s1. Table S1: PRISMA 2020 checklist.

## Abbreviations

The following abbreviations were used in this manuscript:

MRI	Magnetic resonance imaging
DCE	Dynamic contrast enhancement
STS	Soft-tissue sarcoma
LR	Local recurrence
TIC	Time–intensity curve
TCC	Time–concentration curve
EES	Extravascular/extracellular space
rPF	Relative plasma flow
rMTT	Relative mean transit time
iAUC	Initial area under the curve
ROI	Region of interest
SS	Steepest slope
RISMA	Preferred Reporting Items for Systematic Reviews and Meta-Analyses
MeSHs	Medical Subject Headings
PICOS	Patient, Intervention, Comparison Group, Outcome, Study design
DTA	Diagnostic test accuracy
SROC	Summary receiver operating characteristic
3D	Three-dimensional
MPNST	Malignant peripheral nerve sheath tumor
T2WI	T2-weighted imaging
